# Cholinium-Based Ionic Liquids as Promising Antimicrobial Agents in Pharmaceutical Applications: Surface Activity, Antibacterial Activity and Ecotoxicological Profile

**DOI:** 10.3390/pharmaceutics15071806

**Published:** 2023-06-24

**Authors:** María Teresa García, Elena Bautista, Ana de la Fuente, Lourdes Pérez

**Affiliations:** Department of Surfactants and Nanobiotechnology, Institute of Advanced Chemistry of Catalonia (IQAC-CSIC), 08034 Barcelona, Spain; elena.bautista@iqac.csic.es (E.B.); lourdes.perez@iqac.csic.es (L.P.)

**Keywords:** cholinium-based ionic liquids, surface activity, antimicrobial activity, biofilm inhibition, biofilm eradication, hemolysis, biodegradation, ecotoxicity

## Abstract

Cholinium-based ionic liquids are compounds increasingly studied in pharmaceutics and biomedicine to enhance bioavailability in drug delivery systems and as bioactive ingredients in pharmaceutical formulations. However, their potential as antimicrobial agents has scarcely been investigated. Herein, we explored the antimicrobial activity of a series of surface-active cholinium-based ionic liquids (Chol-ILs). For this purpose, Chol-ILs with alkyl chains of 10–16 carbon atoms were synthesized and their self-assembly in aqueous medium was investigated. Subsequently, their antimicrobial activity against a panel of clinically relevant bacteria and their ability to eradicate MRSA and *P. aeruginosa* PAO1 biofilms was evaluated. Finally, we analyzed the ecotoxicological profile of Chol-ILs in terms of susceptibility to aerobic biodegradation and acute aquatic toxicity against *D. magna* and *V. fisheri*. Our results reveal that cholinium-based ILs with alkyl chain lengths ≥12 C show a broad spectrum of antibacterial activity. Their antimicrobial efficacy depends on their hydrophobicity, with the C_14_–C_16_ homologs being the most effective compounds. These ILs exhibit antimicrobial activity similar to that of imidazolium ILs and quaternary ammonium antiseptics. Moreover, the longer alkyl chain Chol-ILs are able to eradicate established biofilms at concentrations as low as 16–32 µg/mL. The biodegradation rate of cholinium-based ILs decreases with alkyl chain elongation. Our results reinforce the suitability of Chol-ILs as promising multifunctional compounds for application in pharmaceutical and biomedical formulation.

## 1. Introduction

Ionic liquids (ILs) are salts mostly composed of bulky N-containing asymmetric organic cations combined with inorganic or organic anions [1]. They have attracted enormous interest in areas such as organic chemistry, catalysis, biomass treatment, separation processes, the preparation of nanostructured materials, etc., due to their excellent chemical and thermal stability, their remarkable capacity to solubilize a wide variety of compounds, and because their characteristics can be finely adjusted by appropriate selection of cation and anion constituents [2,3,4].

Many amphiphilic ionic liquids have emerged as effective surface-active compounds and their aggregation behavior has been extensively studied [4,5,6,7]. Because of their antibacterial and antifungal activities, amphiphilic ILs have been examined for pharmaceutical and biomedical applications [8,9,10]. Moreover, it has been discovered that some ILs enhance drug solubility and/or act as carriers for drug delivery systems [11,12,13,14,15,16]. Therefore, ILs are being investigated for a variety of bioapplications, such as biosensors, protein stabilization, and biocatalysis [17,18,19].

Cationic salts of imidazolium, pyridinium, ammonium, and phosphonium exhibit biocidal activity over a wide range of bacteria and are broadly used as disinfectants in the food and pharmaceutical fields. However, significant drawbacks have already been observed mainly related to their toxicity and persistence [20,21]. Many ILs displayed significant toxicity towards enzymes, microorganisms, and cells, in addition to animals and plants [22,23]; and most are not easily biodegradable [24]. In addition, recent studies have reported the multiplication and dissemination of antibiotic resistance genes by some persistent imidazolium-based ILs because of enhanced horizontal gene transfer, while other similar structures, such as biocidal quaternary ammonium compounds, have long been found to contribute to the rise of antibiotic resistance [25]. 

The use of easily biodegradable ionic liquids could minimize or even avoid the adverse effects of ILs. One possible strategy to decrease accumulation in the environment and the consequent negative impact would be the utilization of cholinium-based ILs as presumably greener compounds in comparison to imidazolium ILs [16]. Cholinium represents an excellent candidate as the IL cation because it is expected to possess comparatively low toxicity. In addition, choline, an essential micronutrient, degrades completely under aerobic conditions [2]. 

The prominent antibiofilm activity described for some structurally related cationic amphiphiles [26] as well as the growing need for new, broad-spectrum, and nonpersistent antimicrobials [20] for biomedical and biotechnological applications has prompted us to investigate the antimicrobial activity and the ability to prevent biofilm formation or eradicate mature biofilms of a series of surface-active choline-derived ionic liquids, as well as the ecotoxicological profile of these compounds in terms of susceptibility to aerobic biodegradation and aquatic toxicity. The results of this research are expected to contribute to the generation of knowledge in the area of the antimicrobial activity and biofilm eradication capacity of cholinium-based ionic liquids that may be of great interest for their application in pharmaceutical and biotechnological applications. At the same time, since the ecotoxicological behavior of surface-active cholinium-based ILs is still little known, the results obtained provide insight into the potential environmental effects of cholinium-based ionic liquids.

## 2. Materials and Methods

### 2.1. Synthesis and Characterization of Cholinium-Based Ionic Liquids (C_n_CholBr)

**Scheme 1:**
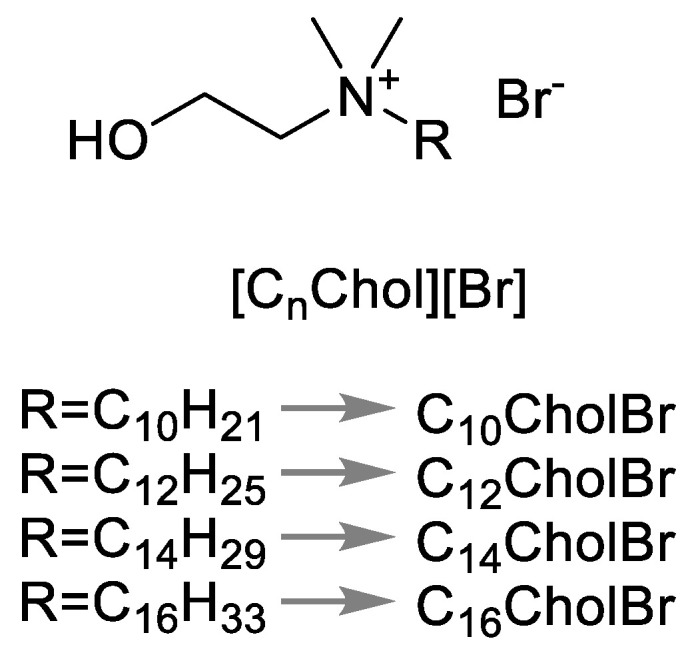
Structure of cholinium-based ionic liquids [C_n_CholBr].

The molecular structure of the cholinium-based ILs (C_n_CholBr) is displayed in Scheme 1. Alkyl(2-hydroxyethyl) dimethylammonium bromide salts were prepared by alkylation of 2-dimehylaminoethanol with the appropriate alkyl halide to afford the corresponding cholinium halide [27]. The synthesis procedure and characterization data of cholinium ILs are given in the Supplementary Materials (Synthesis Section, pages 1 and 2).

### 2.2. Thermal Stability Measurements

Thermogravimetric analyses were performed with a TGA instrument (Model TGA/SDTA 851; Mettler Toledo, Columbus, OH, USA). The instrument was calibrated using indium, zinc, and gold. The analyses were performed under nitrogen purge with a flow rate of 50 mL/min from r.t. to 550 °C at 10 °C/min. Samples were weighted in 100 µL aluminium crucibles using a microbalance (Model XPR2, Mettler Toledo) and sealed with an aluminium lid. The sample was loaded into the furnace by a sample robot (Model TS0801RO, Mettler Toledo) equipped with a lid-piercing accessory, which automatically pierces the lid of the hermetically sealed crucibles immediately before measurement. The diameter of the needle was 100 μm. 

### 2.3. Conductivity Measurements

An Orion Conductivity Cell 913005MD with an epoxy/graphite electrode and a Thermo Orion 5 Star multiparameter instrument with a cell constant of 0.475 cm^−1^ were used to perform conductivity measurements at a temperature of 25 °C [5].

### 2.4. Surface Tension Measurements

Surface tension measurements were made at 25 °C by the Wilhelmy plate technique using a Krüss K-12 tensiometer. The glass containers and plate were carefully rinsed with distilled water after being cleaned with a chromic acid solution. Each measurement was performed with the plate flame-dried [5]. When the standard deviation of five consecutive measurements did not exceed 0.10 mN/m, the surface tension was deemed to be at equilibrium.

### 2.5. Fluorescence Measurements

Steady-state fluorescence measurements were conducted with a Shimadzu RF 540 spectrofluorometer equipped with a thermostated cell holder at 25 °C. Pyrene was used as the fluorescence probe at a concentration of 1 × 10^−6^ M. The fluorescence emission spectra of pyrene dissolved in C_n_CholBr aqueous solutions were recorded from 340 to 450 nm after excitation at 332 nm. The steady-state fluorescence emission spectrum of pyrene shows fine structure in the 370−400 nm region, and the polarity of the environment has a significant effect on the nature and intensity of these bands. To determine the critical micelle concentration (*cmc*) of the ionic liquids in aqueous solution, the ratio of the first to the third vibronic peaks, i.e., I_1_/I_3_, which exhibits the largest solvent dependency, was employed [6,28]. 

### 2.6. Dynamic Light Scattering Measurements

Dynamic light scattering (DLS) measurements were carried out at 25.0 ± 0.1 °C using a light-scattering instrument (Zetasizer, nanoseries, nano-ZS, Malvern Instruments, Malvern, UK) with a He−Ne laser (633 nm, 4 mW) equipped with a built-in temperature controller. For each sample, 30 repeated measurements were taken. From the DLS data, the CONTIN algorithm was used to determine the size distribution of aggregates.

### 2.7. Antimicrobial Activity

Antimicrobial assays were performed against the following microorganisms: *Bacillus subtilis* ATCC 6633, *Staphylococcus epidermidis* ATCC 12228, *Staphylococcus aureus* ATCC 29213, *Listeria monocytogenes* ATCC 15313, *Enterococcus faecalis* ATCC 29212, *Escherichia coli* ATCC 25922, *Acinetobacter baumannii* ATCC 19606, *Klebsiella aerogenes* ATCC 13048, methicillin-resistant *Staphylococcus aureus* ATCC 43300, and *Pseudomonas aeruginosa* PAO1. Minimum inhibitory concentration (MIC) values—the lowest concentration of an antimicrobial agent that prevents the establishment of visible bacterial growth after 24 h at 37 °C—were used to determine the antibacterial activity in vitro. The ionic liquids were dissolved in Mueller−Hinton broth (MBH) at concentrations ranging from 1 to 256 μg/mL [28]. Broth was dispensed (200 μL) in the corresponding wells of a 96-well polypropylene microtiter plate. A total of 10 μL of a nutrient broth starter culture of each bacterial strain was added to achieve final inocula of approximately 5 × 10^−5^ colony-forming units (CFU) per mL. The growth control was nutrient broth medium without the ionic liquid. After incubating the bacteria for 24 h at 37 °C, their growth was visually observed. The increase in cell mass and cell number was reflected by an increase in turbidity [5]. Resazurin was used to confirm the visual MIC values. To this end, 20 μL of resazurin at 0.015% *w*/*v* was added to each well and left to react for approximately 30 min at 37 °C. After the incubation period, the blue-to-pink color change of resazurin was used as an indication of bacterial metabolic activity. All the experiments were performed in triplicate.

### 2.8. Antibiofilm Activity

Antibiofilm activity was assessed against biofilm-producing strains of methicillin-resistant *Staphylococcus aureus* (MRSA) ATCC 43300 and *Pseudomonas aeruginosa* PAO1. The biofilm inhibition and eradication capacities of ILs were evaluated against adherent bacterial biofilms grown in 96-well microtiter plates [29]. Bacteria were grown overnight in Tryptic soy agar at 37 °C for 24 h. The bacteria were suspended in lysogeny broth containing glucose (1%) at 1.5 × 10^8^ CFU/mL.

#### 2.8.1. Biofilm Inhibition Procedure

A total of 100 μL of the ionic liquid at different concentration values was added to each well in a 96-well microplate, then 100 μL of the diluted bacterial suspension was added to the wells. Twofold serial dilutions of the ionic liquids to be tested were used (2–128 μg/mL). Microtiter plates were incubated at 37 °C for 24 h. After treatment, the optical density at 600 nm (OD_600_) was recorded to quantify bacterial growth in each well. Spent growth media was then discarded and the biofilm was fixed with 150 μL methanol for 10 min. The plates were air-dried and the biofilms were stained with 50 μL of 0.1% crystal violet for 25 min. The plates were rinsed with water and then 150 μL of 96% ethanol containing 10% acetic acid was added to all 96 wells to dissolve the crystal-violet-stained biofilm. The absorbance value was read at 570 nm. Each assay was performed at least three times and the results were averaged.

#### 2.8.2. Biofilm Eradication Procedure

A 96-well microplate with 200 µL of the diluted bacterial suspension in each well was used to generate mature biofilms, which were then incubated for 24 h at 37 °C. following a gentle PBS washing, 200 μL of the ionic liquid solution was added to each well and once more incubated at 37 °C for 24 h. Twofold serial dilutions of the ionic liquids to be tested were used (4–256 μg/mL). After treatment, the biofilms were first stained with resazurin for 25 min to estimate bacteria viability [30], and then, the resazurin stain was removed and the biofilm was fixed with 150 μL methanol for 10 min. Finally, CV staining was performed as described at the end of the previous section (Section 2.8.1).

### 2.9. Hemolysis Assay

Fresh blood was drawn from a rabbit to prepare the erythrocyte suspension. Samples of rabbit blood provided by the animal facility of the Research and Development Center (CID)—Spanish National Research Council (CID-CSIC, Barcelona, Spain) were used to isolate erythrocytes. The collection of samples strictly followed the bioethical guidelines outlined by the Spanish legislation. The erythrocytes were washed three times in PBS (pH 7.4). Erythrocytes were then suspended in PBS at a cell density of 8 × 10^9^ cells/mL. We slightly modified the method of Pape et al. [31] to examine cholinium-based ionic liquids for hemolytic activity. Eppendorf tubes holding 25 µL of erythrocyte suspension and 1 mL of PBS were each filled with a variety of quantities of a concentrated IL solution, ranging in size from 10 to 80 µL. After shaking the samples for 10 min at room temperature, the tubes underwent a 5 min, 10,000 rpm centrifugation. By comparing the absorbance (540 nm) of the supernatant of the samples with that of the control, the amount of hemolysis was calculated.

### 2.10. Aerobic Biodegradability

The aerobic biodegradability of the ionic liquids was studied by applying the OECD 310 method (CO_2_ headspace test) [32]. This standard method evaluates the mineralization of an organic compound in an aqueous medium by measuring the production of CO_2_. The rate of biodegradation of cholinium-based ionic liquids was calculated by tracking the CO_2_ produced as these compounds were broken down by microorganisms over time. A mixed population of microorganisms derived from activated sludge from a sewage treatment plant (Manresa, Barcelona) was introduced to each IL solution at a concentration of 10 mg C/L as the sole source of carbon and energy. The reference substance was 20 mg C/L of sodium benzoate (NaBz). Assays of the biodegradation were conducted from 28 to 49 days. Inhibition studies using binary mixtures of NaBz and C_n_CholBr at 20 and 10 mg C/L, respectively, were also carried out for each test ionic liquid. Using a carbon analyzer (Shimadzu TOC-5050), biodegradation was assessed as the net increase in inorganic carbon over time, i.e., the excess of CO_2_ formed in the vessels containing ionic liquid relative to blank containers that monitored the CO_2_ endogenous production. Based on the initial concentration of surfactant, the extent of biodegradation was calculated as a percentage of the theoretical maximum production of inorganic carbon. Since the method’s pass level is set at 60%, a chemical with a higher proportion of biodegradation can be regarded as readily biodegradable.

### 2.11. Aquatic toxicity Tests

#### 2.11.1. *Daphnia magna* Acute Immobilization Test

*D. magna* bioassay [33] was performed to evaluate the adverse effects of the cholinium-based ionic liquids on aquatic biota. The assay used freshwater crustaceans that were laboratory-bred and had not yet reached their 24 h lifespan. The swimming incapability, or the test organism’s inability to move after 15 s of gentle agitation, served as the criterion for effect measurement. The medium had a pH of 8.0 and a Ca/Mg ratio of 4/1, with a total hardness of 250 mg/L (as CaCO_3_). Dark tests were conducted at 20 °C. For each compound, 10 concentrations in a geometric series were evaluated. At each test concentration, 20 daphnia, divided into four groups of five animals each, were used. On a probability–logarithmic scale, the immobility percentage at 48 h was plotted against concentration. The statistical method used to estimate the concentration to immobilize 50% of the daphnia after the exposure period (IC_50_) and the confidence limits (*p* = 0.95) was probit analysis performed with the SPSS statistics software program (IBM SPSS Statistics 27.0.1).

#### 2.11.2. *Vibrio fisheri* Luminescence Reduction Test

*V. fisheri* bioluminescence assay [34] was applied to assess the aquatic toxicity of cholinium-based ionic liquids. When luminescent bacteria were exposed to the tested cationic amphiphiles, the light emission decreased, and this decrease was proportional to the toxicity of the sample. A regression analysis was used to determine the concentration of the compound that caused a 50% reduction (EC_50_) in the amount of light emitted by the bacteria. The stated toxicity statistics were based on bacteria being exposed to the ionic liquid solution for 30 min at 15 °C.

## 3. Results and Discussion

### 3.1. Synthesis of Cholinium-Based Ionic Liquids (C_n_CholBr)

Long alkyl chain cholinium-based ionic liquids (Scheme 1) were synthesized by alkylation of 2-dimethylaminoethanol with the appropriate alkyl halide [27]. The synthesis procedure and characterization data of the cholinium ILs are given in the Supplementary Materials (Synthesis Section, pages 1 and 2). 

### 3.2. Thermal Stability of C_n_CholBr

Thermogravimetric analysis (TGA) was applied to study the thermal stability of the ionic liquids based on cholinium. Thermal weight-loss curves of the cholinium-based ILs under a nitrogen atmosphere are given in the Supplementary Materials (Figure S1). Table 1 displays the decomposition temperature of the investigated cholinium-based ILs. For the sake of comparison, Table 1 also displays the decomposition temperature of imidazolium (1-alkyl-3-methylimidazolium bromides) and pyridinium (1-alkylpyridinium bromides) ILs reported in the literature [5,6].

The cholinium-based ILs are thermally stable up to 238 °C. These organic salts decompose at temperatures 40–50 °C lower than imidazolium ILs and have slightly higher thermal stability than pyridinium ILs (Table 1). Therefore, their range of operating temperatures is similar to that of pyridinium-based ILs. 

### 3.3. Aggregation Behavior in Aqueous Medium

Conductivity, surface tension, and fluorescence measurements were used to investigate the aggregation of cholinium-based ionic liquids in aqueous solution. The sequential steps in the self-assembly of amphiphilic ionic liquids were investigated using these complementary techniques. The information obtained is shown in Table 2 and discussed below.

#### 3.3.1. Conductivity Measurements

**Figure 1:**
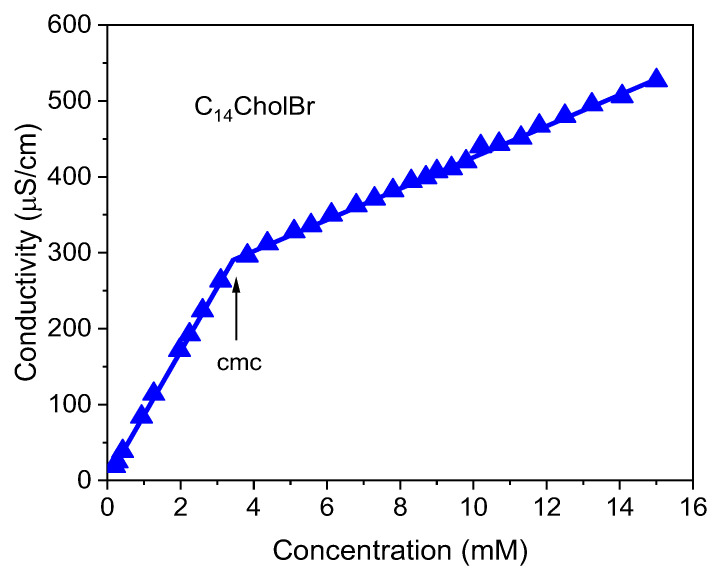
Specific conductivity as a function of C_14_CholBr concentration in water at 25 °C.

The change in the specific conductivity of cholinium-based ILs in relation to their concentration in aqueous solution was examined. Figure 1 shows the characteristic conductometric profile of the C_14_CholBr homolog. Conductometric plots of the rest of the homologs are given in the Supplementary Materials (Figures S2–S4). 

**Figure 2:**
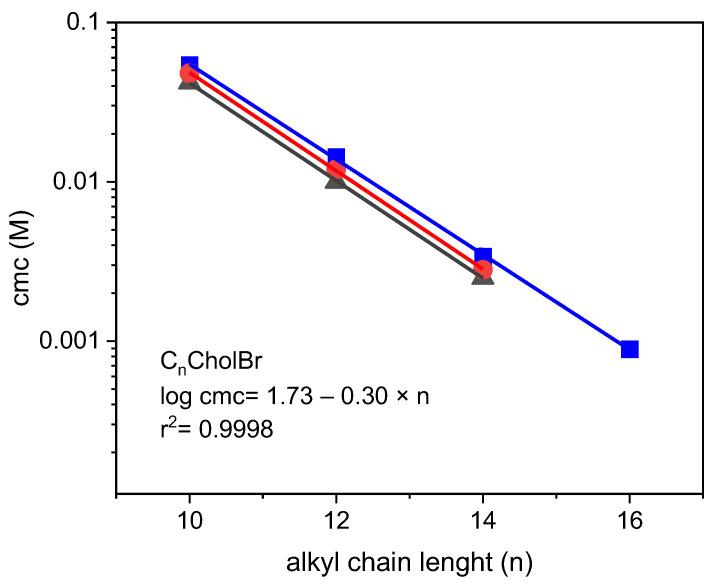
Effect of the alkyl chain length on the *cmc* of C_n_CholBr (■), CnMeImBr (▲), and CnPyrBr (●).

In the pre- and postmicellization zones, the specific conductivity values conform to two straight lines with different slopes. Due to the lower mobility of the micelle compared to the monomers and the binding of some counterions to it, the slope changes abruptly [6]. The *cmc* value was determined from the location of the breakpoint. The degree of ionization, α, is determined by the ratio of the slopes of the linear regions above and below the *cmc*. The degree of binding of the counterion to the micelle, β, is 1-α. The β binding degree estimates the counterions contained in the Stern layer to counteract the electrostatic force opposing self-assembly. The *cmc* values of cholinium-based ionic liquid progressively decrease with the increase in the alkyl chain length (Table 2), as occurs for amphiphilic imidazolium and pyridinium-based ionic liquids [4,35,36]. The *cmc* values for cholinium-based ILs were slightly higher than those reported for imidazolium (1-alkyl-3-methylimidazolium bromides, C_n_MeImBr) and pyridinium (1-alkylpyrinidinium bromides, C_n_PyrBr) ionic liquids [4]. This means that the more polar cholinium head group makes micelle formation slightly less energetically favorable. According to the empirical Stauff–Klevens Equation (1) [37], there is a linear relationship between log *cmc* and alkyl chain length (Figure 2).

(1) logcmc=A−Bxn

where *A* and *B* depend on the characteristics of the polar head group and the impact of each additional methylene group on the *cmc*, respectively, for a specific homologous series [38]. For the cholinium-based ILs (Figure 2), *A* and *B* were found to be 1.7 and 0.30, respectively. These ILs present the characteristic slope values (0.28–0.30) reported for conventional ionic surfactants [38] and imidazolium- and pyridinium-based ILs [4,5,6]. The slope of the linear correlation was used to estimate the free energy change involved in the transfer of one methylene group of the hydrocarbon chain from the aqueous medium to a micelle [38]. This gave a value of −1.71 kJ/mol for the cholinium-based ILs. This value is very close to that described for n-alkyl chain imidazolium ILs (−1.83 kJ/mol) and n-alkyl chain pyridinium ILs (−1.78 kJ/mol).

The counterion binding degree (β) of the cholinium-based ILs (0.61–0.76) (Table 2) is very close to that reported for the long alkyl chain imidazolium and pyridinium bromide salts [4]. The standard Gibbs energy of micellization $\left(\Delta G_{mic}^{o}\right)$ can be estimated from Equation (2) [39] by applying the phase separation model to the monomer–micelle equilibrium for cholinium ILs:

(2) $$\Delta G_{mic}^{0}=\left(1+\beta \right){\mathrm{RT}\;\ln\;x}_{cmc}$$

where β is the binding parameter and $x_{cmc}$ is the *cmc* expressed as a mole fraction. $\Delta G_{cmc}^{o}$ denotes the differences in free energy per mole between ionic liquid molecules in bulk solution and micellar aggregates. The $\Delta G_{cmc}^{o}\;\mathrm{values}$ calculated for cholinium ILs are shown in Table 2. The Gibbs free energy becomes more negative with increasing alkyl chain length, demonstrating the existence of hydrophobic interactions governing micelle formation.

#### 3.3.2. Surface Tension Measurements

**Figure 3:**
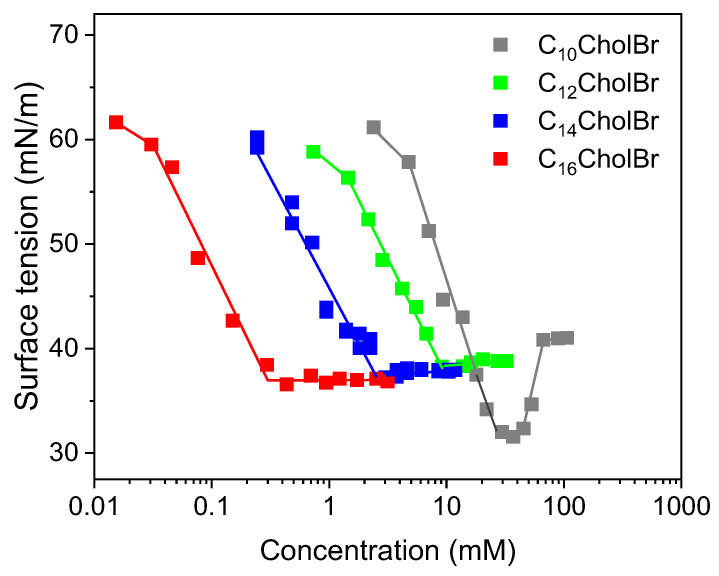
Plot of surface tension vs. concentration for C_n_CholBr in water at 25 °C.

Surface tension measurements were used to study the surface activity of C_n_CholBr. Figure 3 displays the plots of surface tension (γ) vs. the logarithm of concentration for the cholinium-based ILs in aqueous solution at 25 °C.

The surface tension of the solution decreases progressively until a concentration of IL in the medium is reached at which stabilization occurs (Figure 3). In the case of C_10_CholBr, a marked surface tension minimum is detected before reaching a plateau. The presence of such a minimum has already been reported for 1-alkyl-3-methylimidazolium salts and amide- and carbonate-functionalized imidazolium ILs having alkyl chains of 8-10 carbon atoms [4,5,6,35,40]. The existence of such a minimum on the surface pressure curve has been tentatively ascribed to the presence of surface micelles before bulk assembly and the re-establishment of a surface monolayer at concentrations above the *cmc* [36]. The *cmc* values for cholinium-based ILs based on surface tension measurements (Table 2) decrease with increasing alkyl chain length and agree well with those determined by conductometry. 

The efficiency of adsorption, *p*C_20_ (defined as the negative logarithm of the concentration of amphiphilic molecules required to reduce the surface tension of the pure solvent by 20 mN/m), and the surface tension reduction effectiveness, *Π_cmc_* (defined as *Π_cmc_* = *γ*_0_ − *γ_cmc_*, where *γ*_0_ is the surface tension of pure water), were also determined using surface tension isotherms. The pC_20_ and Π*_cmc_* values of the cholinium-based ILs are displayed in Table 2. The adsorption efficiency (pC_20_) of the investigated ionic liquids (Table 2) increases linearly with the alkyl chain length, i.e., the cholinium-based ionic liquids adsorb more strongly at the interface and more efficiently reduce surface tension with increasing hydrophobicity. The investigated cholinium-based ILs exhibit similar values for efficiency of adsorption and effectiveness (Table 2) to those reported for 1-alky-3-methylimidazolium and 1-alkylpyridinium bromide salts [4,41], which means that cholinium-derived ILs have a comparable surface activity to imidazolium- and pyridinium-derived ILs. 

Surface tension vs. concentration data were subjected to the Gibbs adsorption isotherm equation to determine the mean area per molecule present at the surface, A_min_ [38]. A_min_ slightly increases with alkyl chain length, suggesting that the molecules of cholinium-based IL are arranged in a slightly looser arrangement at the air–solution interface. From Equation (3), the standard free energy of adsorption (Δ*G^o^_ads_*) was calculated (3) [38]:

(3) $$\Delta G_{ads}^{o}=\Delta G_{mic}^{o}-\frac{\Pi_{cmc}}{\Gamma_{max}}$$

where Γ_max_ is the surface excess concentration. The Δ*G^o^_ads_* values (Table 2) become more negative with the elongation of the alky chain of the cholinium-based ILs. The higher magnitude of Δ*G^o^_ads_* compared to Δ*G^o^_mic_* reveals that adsorption at the air–water interface is more favored than micellization in the bulk for these amphiphilic compounds. 

#### 3.3.3. Fluorescence Measurements

**Figure 4:**
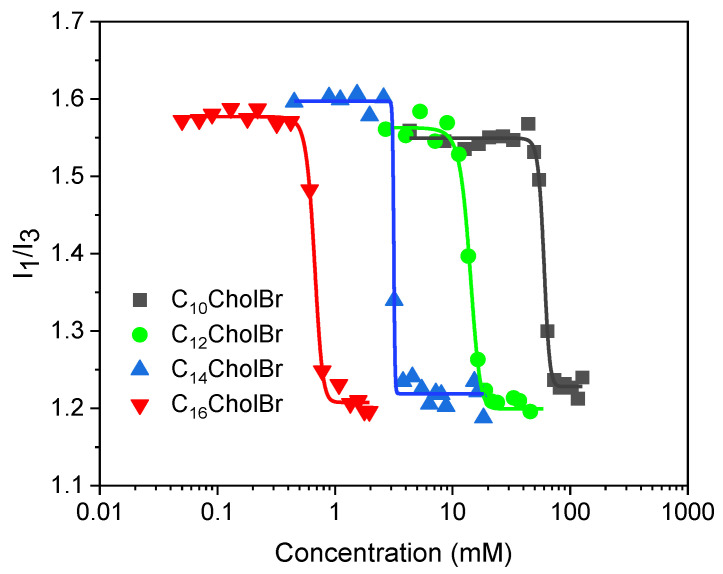
Change in the intensity ratio I_1_/I_3_ of pyrene vs. IL concentration in water at 25 °C.

The aggregation properties of the cholinium-based ILs were also determined by fluorescence measurements using pyrene as a solvatochromic probe. The polarity of the fluorescence probe’s microenvironment was monitored as a function of IL concentration, and the ratio of the intensity of the first (I_1_) to the third (I_3_) vibronic peaks of pyrene was measured (Figure 4). The sharp reduction in I_1_/I_3_ intensity provides evidence that IL aggregates have formed. The *cmc* value was calculated as the midpoint of the transitions since the data fit well to sigmoidal Boltzmann-type curves.

The *cmc* values by fluorescence measurements (Table 2) are similar to those obtained from the conductivity and surface tension measurements and confirm the decrease in *cmc* values with increasing hydrophobicity of the IL.

#### 3.3.4. Size of Micellar Aggregates

**Figure 5:**
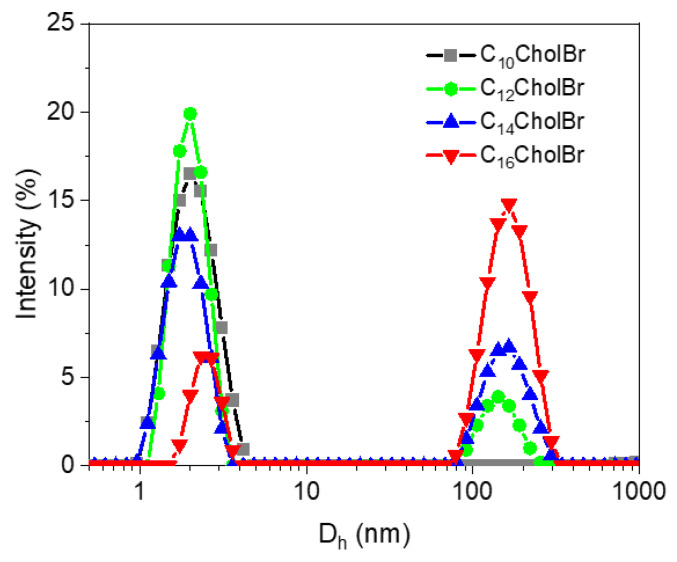
Intensity size distribution profiles for aggregates of cholinium-based ILs.

The aggregate size of cholinium-based ILs in water was measured by dynamic light scattering (DLS). DLS analyses to determine the hydrodynamic diameter (D_h_) of the aggregates were performed at concentrations 3–5 times greater than the *cmc* values, namely, 162, 43, 20, and 4.5 mM for the C_10_, C_12_, C_14_, and C_16_ homologs, respectively. Figure 5 shows the intensity size distribution profiles obtained for the ILs studied.

For the C_10_CholBr homolog, a size distribution with a hydrodynamic diameter, D_h_, was observed with a maximum scattering intensity at 2.0 nm (Figure 5). For the rest of the C_n_CholBr homologs (C_12_ to C_16_), the intensity distribution profiles (Figure 5) display two size distributions, one with a hydrodynamic diameter, D_h_, with a maximum scattering intensity in the range of 2.0–2.5 nm and the other with D_h_ in the range of 145–170 nm. Lower D_h_ values have been attributed to micelle diameter, whereas larger D_h_ values could be attributed to micellar agglomerates [42]. The number-weighted size distributions (Figure S5, Supplementary Materials) indicate that the number of small micelles prevails over that of micellar agglomerates. The results suggest the spontaneous formation of small micelle-like aggregates during the self-assembly of cholinium-based ionic liquids. 

### 3.4. Antimicrobial and Antibiofilm Activity

In the search for new safe and effective antimicrobials, significant interest has been focused on the development of bio-based ionic liquids to substitute the environmentally questioned imidazolium-based ionic liquids.

#### 3.4.1. Antimicrobial Activity

The antimicrobial activity of cholinium-based ILs was assessed against a range of representative Gram-positive and Gram-negative bacteria by determining their minimum inhibitory concentration (MIC), i.e., the concentration of ionic liquid required to completely inhibit the growth of the microorganisms. Table 3 displays the MIC values for the different alkyl chain homologs of C_n_CholBr. For comparison, MIC values were also determined for a conventional imidazolium-based ionic liquid (C_14_MeImBr) and one of the most commonly used quaternary ammonium antiseptics (benzalkonium chloride, BAC) (Table 3).

The antimicrobial activity of cholinium-derived ionic liquids depends on the length of their alkyl chain (Table 3). Increasing the number of carbon atoms in the alkyl chain from 10 to 16 leads to a dramatic increase in the antimicrobial efficacy of these ionic liquids. Thus, the most hydrophilic compound of the series, C_10_CholBr, shows low antimicrobial activity against Gram-positive bacteria and negligible activity against Gram-negative bacteria. Cholinium-based ionic liquids with alkyl chains ≥12 C exhibit a broad spectrum of antimicrobial activity against Gram-positive and Gram-negative bacteria. Their efficacy as antimicrobial agents increases with alkyl chain elongation, reaching maximum values for the C_14_–C_16_ homologs. These compounds are more active against Gram-positive bacteria than Gram-negative bacteria. The lower activity of cholinium-based ILs against Gram-negative bacteria may be ascribed to the lower permeability of their outer membranes [43]. 

When the MIC values of the cholinium-based ILs (Table 3) are compared to their *cmc* values (16,757, 4737, 1246, and 351 expressed as µg/mL for the C_10_, C_12_, C_14_, and C_16_ homologs, respectively), it is clear that self-aggregation processes occur at concentrations that are higher than those required for antimicrobial activity. This indicates that the toxicity of cationic amphiphiles against bacteria is mainly exerted by monomeric species.

Comparing the antimicrobial activity of C_14_–C_16_ choline-based ILs with that of imidazolium-derived ionic liquids (C_14_MeImBr) and quaternary ammonium antiseptics (BAC) (Table 3), it is observed that all these cationic amphiphiles exhibit a broad spectrum of antibacterial activity. Moreover, the antimicrobial efficacy of cholinium-based ILs is comparable to that of imidazolium-based ILs and QACs with similar alkyl chain lengths. This may be attributed to a common mode of antimicrobial action of cationic amphiphiles against bacteria, which mainly involves cell membrane disruption [44,45]. Thus, cationic amphiphiles attach to the bacterial cell membrane through ionic and hydrophobic interactions, altering the characteristics and function of the membrane in the process [46]. 

#### 3.4.2. Antibiofilm Activity

When bacteria grow on surfaces, they typically create biofilms. Biofilms are estimated to be responsible for 65% of microbial infections in humans [47]. Biofilms enable bacterial tolerance to environmental threats and also promote the transfer of antimicrobial resistance genes between bacterial species [48]. Therefore, it is crucial to develop new antimicrobials capable of inhibiting biofilm growth and/or eradicating mature biofilms. 

**Figure 6:**
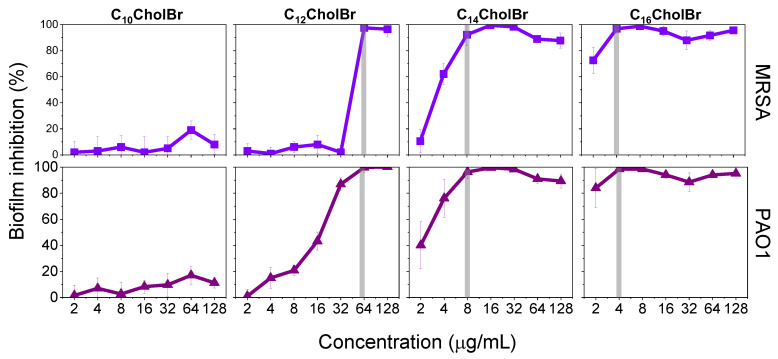
Biofilm inhibition of cholinium-based ILs vs. MRSA ATCC 43300 (■) and *P. aeruginosa* PAO1 (▲) based on CV staining. Calculations were made in relation to untreated bacteria (defined as 100%) and sterile control wells (defined as 0%) to determine the percentage of biofilm inhibition. Data points are the average (±SD) of at least three independent replicates. Based on OD_600_ measurements taken before CV staining, vertical grey lines show the concentration at which more than 90% of bacterial growth is suppressed.

To evaluate the ability of the investigated cholinium-based ionic liquids to inhibit MRSA and PAO1 biofilm formation, C_n_CholBr samples were added to freshly prepared bacterial suspensions and then incubated at 37 °C for 24 h. After this time, bacterial growth was quantified by measuring absorbance at 600 nm and CV staining was applied to measure biofilm biomass. The results are displayed in Figure 6. 

The results of the biofilm inhibition assays (Figure 6) indicated that biofilms formed by both MRSA and PAO1 exhibited similar profiles when in contact with cholinium-based ILs. The antibiofilm activity of these ionic liquids is highly dependent on their hydrophobicity. Thus, the homolog with the shortest alkyl chain length, C_10_CholBr, showed no antibiofilm activity, whereas the antibiofilm activity of the C_12_ to C_16_ homologs increased significantly with alkyl chain length, reaching the maximum activity for C_16_CholBr. The cholinium-based ILs with alkyl chains ≥12 C strongly inhibited biofilm formation at concentrations equal to or greater than those that prevented bacterial growth under test conditions (Figure 6).

**Figure 7:**
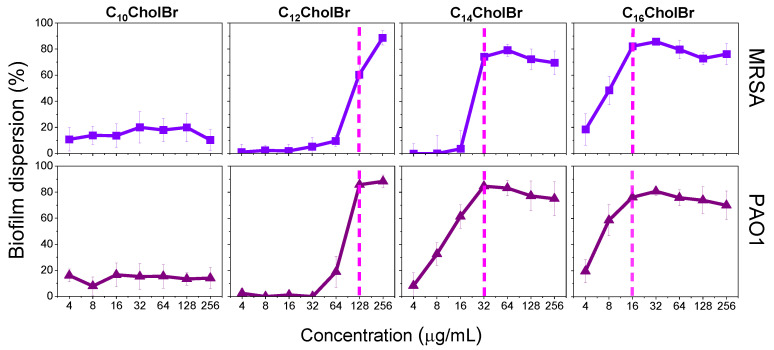
Results of biofilm eradication assays with cholinium-based ionic liquids vs. MRSA ATCC 43300 and P. aeruginosa PAO1. The removal of MRSA (■) and PAO1 (▲) biofilm biomass was quantified by CV staining while the metabolic activity of the biofilms was assessed by resazurin staining (dashed pink vertical lines indicate the lowest concentration of ionic liquid at which there is no bacterial metabolic activity). Results displayed are the mean (±SD) of at least three independent replicates.

To evaluate the capacity of the C_n_CholBr homologs to act on established MRSA and PAO1 biofilms, eradication assays were performed on microtiter plates. The applied method combined the use of the metabolic dye resazurin to assess residual viable metabolizing bacteria and CV staining to measure biofilm biomass. The results are shown in Figure 7.

The results of the biofilm elimination assays showed that C_n_CholBr compounds behaved similarly against MRSA and PAO1 biofilms and that their biofilm eradication ability was essentially dependent on their alkyl chain length (Figure 7). C_10_CholBr showed no biofilm eradication activity, whereas the C_12_–C_16_ homologs were able to efficiently disperse preformed biofilms (75–90%) in a dose-dependent manner. The longer homologs, C_14_CholBr and C_16_CholBr, showed a high antibiofilm effect at low concentrations (Figure 7). Thus, C_14_CholBr and C_16_CholBr are able to remove >80% of the adhered biomass at concentrations as low as 32 and 16 µg/mL, respectively. The concentration of ionic liquid required to eradicate preformed biofilms (Figure 7) is about four times higher than that required to inhibit biofilm formation (Figure 6), which corroborates the greater difficulty of treating bacterial biofilms with respect to planktonic bacteria [47,48].

Evaluation of cell viability in preformed MRSA and PAO1 biofilms treated with cholinium-based ionic liquids revealed that metabolic activity is completely inhibited at the same concentration at which maximum reduction in adhered biomass occurs, indicating that cholinium ILs are capable of simultaneously dispersing biofilm biomass and effectively killing bacterial cells. As occurs with other cationic amphiphiles, such as quaternary ammonium surfactants [26], the antibiofilm activity of these ionic liquids can be attributed to their cationic nature and the presence of hydrophobic alkyl chains. The suggested mechanism of action for the elimination of biofilms by cationic amphiphiles entails the disruption of the extracellular polymeric matrix by electrostatic interactions that disperse the biofilm and cause bacterial cell death. The efficiency of the investigated cholinium-based ionic liquids in eradicating MRSA and PAO1 biofilms is comparable to that described for quaternary ammonium compounds against Gram-positive biofilms [26].

### 3.5. Hemolytic Activity

**Figure 8:**
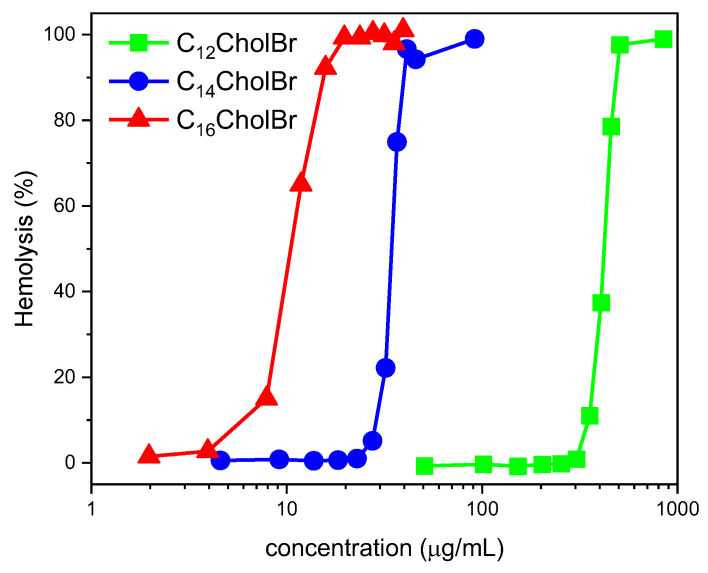
Dose–response curves for the hemolytic activity of C_n_CholBr.

The assessment of cytotoxicity is of great interest in establishing the potential use of antimicrobial compounds in biomedical and pharmaceutical applications. In this work, hemolysis has been used for an initial assessment of cell toxicity. The hemolytic activity of cholinium-based ionic liquids was determined in erythrocytes from rabbit blood samples employing the standard methodology. Hemoglobin release was used to evaluate hemolytic activity as a function of ionic liquid concentration. The obtained dose–response curves are displayed in Figure 8.

The hemolytic activity of C_n_CholBr grows exponentially with IL concentration (Figure 8). The plots of the percentages of hemolysis vs. IL concentration (Figure 8) were used to calculate the concentration that causes hemolysis in 50% of erythrocytes. Table 4 summarized the HC_50_ values.

HC_50_ values increase significantly with the length of the hydrocarbon chain of the IL (Table 4), demonstrating that the hydrophobic character of the ionic liquid plays a key role in its cellular toxicity against erythrocytes. This trend is consistent with that generally described in the literature of increasing hemolytic effect with the alkyl chain length for other cationic amphiphiles such as amino acid-based cationic surfactants [49]. 

The therapeutic use of antimicrobial drugs depends on their ability to selectively destroy bacterial cells without significantly damaging mammalian cells. The selectivity of cholinium-based ionic liquids was determined by means of the therapeutic index [49] expressed as the HC_50_/MIC ratio (Table 5). The higher the value of the therapeutic index, the higher the selectivity of the antimicrobial compound.

The therapeutic index values shown in Table 5 demonstrate that C_12_CholBr and C_14_CholBr possess antibacterial activity against Gram-positive bacteria at concentrations significantly lower than those at which they exhibit erythrocyte toxicity (Table 5). C_12_CholBr is more selective than C_14_CholBr, although the latter clearly exhibits greater antibacterial activity. In the case of C_12_CholBr, a wide safe concentration range was observed against *B. subitilis, S. epidermidis, S. aureus*, and *E. faecalis*, with HC_50_ values between 13 to 52 times higher than MIC values, while it was moderate against *L. monocytogenes* and MRSA, with HC_50_ values 3-to-6-fold higher than MIC values. C_12_CholBr was also selective against some Gram-negative bacteria such as *E. coli* and PAO1 (HC_50_ values between 6 and 7 times higher than MIC values). C_14_CholBr behaved as an effective antibacterial agent (Table 3) and exhibited high selectivity against most of the Gram-positive bacteria investigated (therapeutic index values ranged from 10 to 20 for *B. subtilis, S. epidermidis, S. aureus*, and *E. faecalis*) and moderate selectivity against *L. monocytogenes* and MRSA (therapeutic index values ranged from 2.5 to 5). C_14_CholBr also showed selectivity against some Gram-negative bacteria (*E. coli* and PAO1). In the case of C_16_CholBr, although this cationic amphiphile has similar or even higher antibacterial activity than C_14_CholBr, the higher hemolytic activity of this compound results in the lowest therapeutic indexes against all tested microorganisms. 

Overall, the therapeutic index of cholinium-based ionic liquids is significantly broader against most Gram-positive bacteria than against Gram-negative bacteria (Table 5). This means that, although surface-active cholinium-based ionic liquids efficiently disrupt Gram-positive bacterial membranes (Table 3), they do not significantly impair erythrocyte membranes above their respective MICs (Table 5). Differences in lipid composition and surface charge between bacterial and erythrocyte membranes are essential for cell selectivity. The surface of Gram-positive bacteria is abundant in negatively charged lipids, such as phosphatidylglycerol and teichoic acids, whereas the surface of red blood cells is less negatively charged [49,50]. This leads to stronger electrostatic interactions between the bacteria and cationic amphiphiles than with erythrocytes, resulting in strong antimicrobial activity, but relatively low hemolytic activity. Our results demonstrate that cholinium-based ionic liquids possess a sufficiently broad therapeutic range to be useful as nonhemolytic antimicrobial agents in specific applications.

### 3.6. Aerobic Biodegradability

Although ILs are interesting for a wide variety of applications owing to their chemical and thermal stability, this stability could lead to low biodegradability, which would imply the risk of persistence in the environment [20]. Therefore, knowledge of the biodegradability of cholinium-based ionic liquids is essential to assess their environmental profile. The aerobic biodegradation of cholinium-based ionic liquids was evaluated by applying the CO_2_ Headspace Test (OECD 310). The degree of biodegradation was expressed as a percentage of the theoretical maximum formation of inorganic carbon based on the initial ionic liquid concentration. Table 6 shows the biodegradation percentages of the cholinium-based ionic liquids.

**Figure 9:**
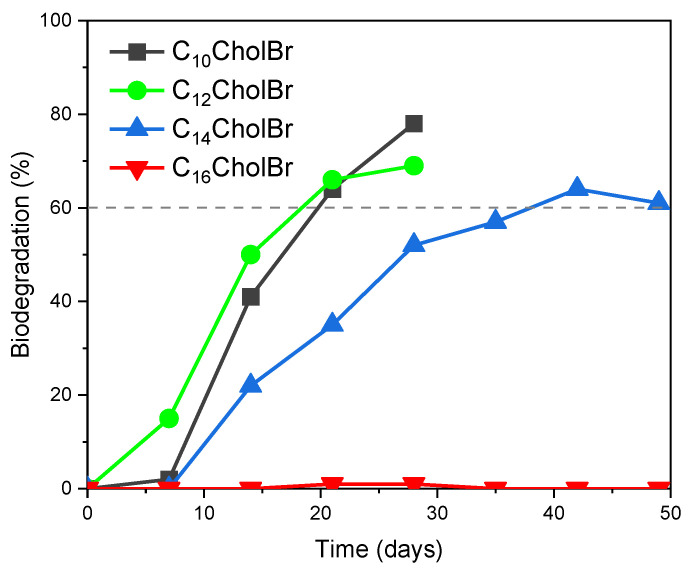
Biodegradation curves of C_n_CholBr by the CO_2_ headspace test.

Significant differences in biodegradation profiles are observed as a function of the alkyl chain length of the cholinium-based ionic liquid (Figure 9). The C_10_ to C_14_ homologs underwent a high degree of mineralization at the end of the 28-day period (Table 7), while C_16_CholBr showed zero biodegradation under the test conditions. The shorter homologs, C_10_ and C_12_CholBr, exceeded the threshold value established in the biodegradation test (>60%) and can be considered easily biodegradable compounds. C_14_CholBr underwent significant mineralization at 28 days (Table 6, 52% biodegradation) but did not attain the pass level of the test. When the assay was continued for a further three weeks, the mineralization of the compound exceeded 60% (Figure 9). This means that C_14_CholBr is inherently biodegradable, but its biodegradation kinetics are slower than the shorter C_10_–C_12_ homologs. C_16_CholBr showed zero biodegradation even over a prolonged period of time. Since the biocidal activity previously shown by some of these ionic liquids could negatively affect their biodegradation, tests were carried out to check the possible inhibitory activity of these compounds against bacterial inoculum. The toxicity of the ionic liquids to the biodegradation inoculum was evaluated by determining the inhibition of the biodegradation of the reference substance (sodium benzoate) in the presence of the investigated ionic liquid (Table 7).

While cholinium-based ionic liquids with alkyl chains ≤ 14 do not exert inhibitory effects on the activity of the bacterial population, C_16_CholBr, the most hydrophobic compound of the series, strongly inhibits the activity of bacteria responsible for ultimate biodegradation. Therefore, the nonbiodegradation of C_16_CholBr is clearly attributable to its biocidal effects on aerobic microorganisms under the test conditions. Since the investigated cholinium-derived ionic liquids have a common structure and differ only in the length of the hydrocarbon chain, it would be expected that C_16_CholBr at sufficiently low (sub-inhibitory) concentrations could be mineralized by the same mechanisms involved in the biodegradation of the shorter homologs. This assumption cannot be corroborated in this type of biodegradation test because the characteristics of the test do not allow working with very low concentrations, which in many cases are the most realistic due to the dilution that occurs in wastewater collectors. Likely, under real conditions, concentrations will be much lower and the toxic effects reduced, so that degradation of the compound can be expected to occur. 

Our results are consistent with those reported in the literature on the biodegradation of cholinium-based ILs that describe these compounds as readily biodegradable [2,51,52] and others that report a significant biodegradation extent for cholinium-based ILs with longer alkyl chains [27]. Unlike imidazolium-based ILs, which have proven to be highly resistant to biodegradation due to the difficulty of cleaving the heterocyclic ring present in these compounds or their metabolites [20,24,53,54,55], cholinium-based ionic liquids undergo rapid and complete mineralization for alkyl chain homologs below 16 carbon atoms. 

### 3.7. Aquatic Toxicity

To assess the ecotoxicity of cholinium-based ionic liquids, toxicity tests were performed on freshwater crustacea (*D. magna*) and saltwater bacteria (*V. fisheri*). *Daphnia magna* is a planktonic crustacean very sensitive to pollution that is widely used to assess surfactant toxicity [38,39,40]. The toxicity of ILs on *Daphnia magna* was evaluated by taking as an effect criterion the immobility of these daphnids after 48 h exposure at different IL concentrations. The bioluminescent inhibition assay with the marine bacterium *Vibrio fisheri* is a highly standardized and also broadly used bioassay. The bioluminescence reduction was measured after 30 min of exposure to increasing concentrations of ionic liquids to determine aquatic toxicity in marine bacteria. The results of the 48 h immobilization test of *D. magna* and the 30 min luminescence reduction test of *V. fisheri* are given in Table 8. 

The concentration of ionic liquid that immobilized 50% of the freshwater crustacean population (IC_50_) ranged from 0.12 to 0.76 mg/L, while the concentration that reduced the luminescence of the bacteria to 50% (EC_50_) ranged from 2.8 to 0.60 mg/L. Although toxicity values are quite similar for both species, daphnia appears to be slightly more sensitive to the action of ionic liquids (Table 8). Based on the IC_50_ values, the investigated cholinium-based ionic liquids belong to the class of chemicals considered “very toxic to aquatic life (Acute toxicity I)” according to the Ecotoxicity Hazard classes proposed by the OECD [56], as their IC_50_ values are less than 1 mg/L.

**Figure 10:**
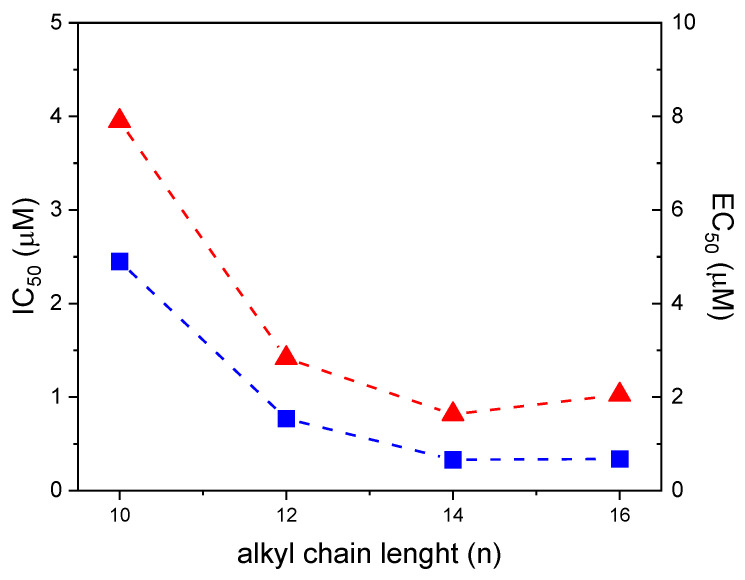
Aquatic toxicity of C_n_CholBr against freshwater crustacean (*D. magna*) (■) and luminescent saltwater bacteria (*V. fisheri*) (▲).

When plotting the toxicity values against the alkyl chain length of the ionic liquid, toxicity increases (lower IC_50_ and EC_50_ values) with increasing alkyl chain length from 10 to 14 carbon atoms, while toxicity is maintained or slightly decreased for the C_16_CholBr (Figure 10).

This demonstrates that cation hydrophobicity plays the main role in the aquatic toxicity of cholinium-based ionic liquids. The tendency for stabilization or a slight decrease in toxicity for ILs having more than 14 carbon atoms in the alkyl side chain could be attributed to the lower bioavailability of the longer alky chain counterparts due to their lower solubility [57]

Compared to cholinium-based ionic liquids with short alkyl chain substituents such as cholinium acetate and cholinium chloride [58], the toxicity of the cholinium-based ILs with long alkyl chains (C_10_ to C_16_) is about 1000 to 10,000 times greater against freshwater crustacea and between 170 to 1100 times higher against luminescent marine bacteria. This supports the strong correlation of toxicity with the alkyl side-chain length of ionic liquids and means that hydrophobicity determines not only the antimicrobial efficacy of these compounds (Section 3.4) but also their aquatic toxicity. 

Choline-based cationic amphiphiles exhibit similar toxicity to that of conventional cationic surfactants, such as alkyl trimethyl ammonium salts and alkyl benzyl dimethyl ammonium [57], and are more toxic than common amphoteric and anionic surfactants [59,60]. Compared to conventional imidazolium-based ILs, the aquatic toxicity of the cholinium ILs is clearly lower than that reported for imidazolium salts with the same alkyl chain length. 1-dodecyl-3-methylimidazolium chloride, C_12_MeImCl, and 1-hexadecyl-3-methylimidazolium chloride, C_16_MeImCl, presented IC_50_ values against D. magna of 0.0043 and 0.0034 mg/L, respectively [61], being between 60 and 38 times more toxic than the corresponding cholinium salts. As a result, the use of cholinium-based ILs could be less harmful to the environment than that of imidazolium-based ILs.

## 4. Conclusions

Greener bioactive ionic liquids based on choline as the cationic scaffold have been synthesized and their physicochemical and biological properties have been investigated. Amphiphilic cholinium-based ILs form micellar aggregates in aqueous solution and exhibit a surface activity comparable to imidazolium- and pyridinium-based ILs. Their tendency to self-assemble increases with the lengthening of the alkyl chain linked to the polar head group. 

Cholinium-based ILs show a broad spectrum of antibacterial activity, being more active against Gram-positive bacteria than against Gram-negative bacteria. Their efficacy as antimicrobial agents depends on their alkyl chain length. Homologs with alkyl chains lower than 12 carbon atoms are not active against bacteria while C_12_–C_16_ homologs display prominent antibacterial activity with C_14_–C_16_ homologs being the most efficient antimicrobial agents. The antimicrobial potency of cholinium-based ILs is similar to that of imidazolium-based ILs with the same alkyl chain length and widely used quaternary ammonium disinfectants such as benzalkonium chloride. Amphiphilic cholinium-based ionic liquids have been found to be potent eradicators of bacterial biofilms. Thus, C_14_–C_16_ homologs are able to effectively eradicate pre-established MRSA and PAO1 biofilms at low concentrations (≤32 µg/mL) simultaneously producing biofilm dispersion and cell death. Moreover, cholinium-based ionic liquids exhibit good selectivity for Gram-positive bacterial cells over mammalian cells. 

Cholinium ILs with alkyl chains shorter than 14 carbon atoms are readily biodegradable, achieving more than 60% biodegradation in 28 days. C_14_CholBr can be considered inherently biodegradable, as evidenced by the fact that it exceeds 60% mineralization, however, it exhibits slower degradation kinetics than shorter alkyl chain homologs. C_16_CholBr did not pass the test for ready biodegradability, which has been proven to be attributable to its toxicity against bacterial inoculum. The aquatic toxicity of C_n_CholBr increases with increasing hydrophobicity, but some stabilization occurs for the longer homologs due to their lower availability in the aqueous medium.

Cholinium-based ILs emerge as new biodegradable antimicrobial agents with a broad spectrum of antibacterial activity and high biofilm eradication capacity. These cationic amphiphiles exhibit a similar antimicrobial activity to imidazolium-based ILs but a clearly improved ecotoxicological profile, being more biodegradable and less toxic, making them promising antimicrobial agents for pharmaceutical and biomedical applications. 

## Figures and Tables

**Table 1:** Thermal decomposition temperatures (T_onset_) of ionic liquids.

ILs	Alkyl Chain Length	T_onset_ (°C)
Cholinium ILs	C_10_–C_16_	238–241
Imidazolium ILs	C_10_–C_14_	282–288
Pyridinium ILs	C_12_	232

**Table 2:** Aggregation parameters of cholinium-based ionic liquids in aqueous solution at 25 °C.

C_n_CholBr	*cmc* ^a^ (mM)	*cmc* ^b^ (mM)	*cmc* ^c^ (mM)	β	ΔG^o^_mic_ (kJ/mol)	π*_cmc_* (mN/m)	pC_20_	A_min_ (nm^2^)	ΔG^o^_ads_ (kJ/mol)
C_10_CholBr	54	49	59	0.61	−29.2	31.2	2.1	-	-
C_12_CholBr	14	9.1	14	0.69	−36.6	33.5	2.7	86	−54.0
C_14_CholBr	3.4	2.5	3.2	0.76	−42.2	34.6	3.3	89	−60.8
C_16_CholBr	0.89	0.35	0.67	0.72	−47.1	35.1	4.2	91	−66.3

Critical micelle concentration (*cmc*) values from ^a^ conductivity, ^b^ surface tension, and ^c^ fluorescence measurements, binding degree (β), Gibbs free energy of micellization (ΔG^o^_mic_), adsorption effectiveness (π*_cmc_*), efficiency of adsorption (pC_20_), area per molecule (A_min_) and standard free energy of adsorption (ΔG^o^_ads_). The values of *cmc* obtained from conductivity, surface tension, and fluorescence were found to have coefficients of variation of ≤7%, ≤10%, and ≤6%, respectively.

**Table 3:** Antimicrobial activity expressed as MIC values.

Microorganism	MIC Values (μg/mL)					
	C_10_CholBr	C_12_CholBr	C_14_CholBr	C_16_CholBr	C_14_MeImBr	BAC
*B. subtilis*	256	16	2	4	2	2
*S. epidermidis*	64	8	2	1	1	2
*S. aureus*	256	32	4	2	2	2
*L. monocytogenes*	>256	128	16	16	16	16
*E. faecalis*	128	32	4	4	2	2
*E. coli*	>256	64	16	32	16	16
*A. baumannii*	>256	256	64	16	16	16
*K. aerogenes*	>256	>256	64	32	32	16
*MRSA*	>256	64	8	4	4	4
*PA01*	>256	64	8	4	4	4

**Table 4:** Hemolytic activity of cholinium-based ionic liquids.

Cationic Amphiphile	HC_50_ (μg/mL)
C_12_CholBr	420 ± 2
C_14_CholBr	34 ± 0.2
C_16_CholBr	11 ± 0.1

**Table 5:** Therapeutic index of cholinium-based ionic liquids.

	Therapeutic Index (HC_50_/MIC)		
	C_12_CholBr	C_14_CholBr	C_16_CholBr
*B. subtilis*	26	20	2.7
*S. epidermidis*	52	20	11
*S. aureus*	13	10	5.4
*L. monocytogenes*	3.3	2.5	0.7
*E. faecalis*	13	10	2.7
*E. coli*	6.6	2.5	0.3
*A. baumannii*	1.6	0.6	0.7
*K. aerogenes*	-	0.6	0.3
*MRSA*	6.6	5.0	2.7
*PA01*	6.6	5.0	2.7

**Table 6:** Biodegradation study (CO_2_ Headspace test) of cholinium-based ionic liquids. Percentages of biodegradation of ionic liquids and the reference substance (NaBz).

Compound	Biodegradation (%)			
	7 Days	14 Days	21 Days	28 Days
NaBz	81 ± 9.2	89 ± 7.1	93 ± 5.8	91 ± 5.3
C_10_CholBr	2 ± 2.9	41 ± 12.3	64 ± 11.4	78 ± 5.7
C_12_CholBr	15 ± 4.2	50 ± 13.2	66 ± 12.7	69 ±1.8
C_14_CholBr	0 ± 0.7	22 ± 13.1	35 ± 11.8	52 ± 4.9
C_16_CholBr	0 ± 0.1	0 ± 0.1	1 ± 0.9	0 ± 0.1

**Table 7:** Biodegradation inhibition of sodium benzoate by cholinium-based ionic liquids.

Mixture	Concentration (µg C/mL)	Inhibition (%)
NaBz + C_10_CholBr	20 + 10	0
NaBz + C_12_CholBr	20 + 10	0
NaBz + C_14_CholBr	20 + 10	0
NaBz + C_16_CholBr	20 + 10	85 ± 6

**Table 8:** Toxicity results of C_n_CholBr in the *Daphnia magna* acute immobilization assay and the *Vibrio fisheri* luminescence inhibition assay expressed as IC_50_ and EC_50_ concentrations, respectively, with their 95% confidence intervals.

Ionic Liquid	*D. magna*, IC_50_ (95% CI) (mg/L)	*V. fisheri*, EC_50_ (95% CI) (mg/L)
C_10_CholBr	0.76 (0.59–0.95)	2.8 (2.0–3.9)
C_12_CholBr	0.26 (0.23–0.29)	0.96 (0.94–0.98)
C_14_CholBr	0.12 (0.12–0.13)	0.60 (0.27–0.71)
C_16_CholBr	0.13 (0.12–0.15)	0.81 (0.61–1.1)

## Data Availability

The data presented in this study are available within this article.

## Supplementary Materials

The following supporting information can be downloaded at: https://www.mdpi.com/article/10.3390/pharmaceutics15071806/s1, Synthesis section, pages 1 and 2; Figure S1: Decomposition curves determined by TGA for C_n_CholBr; Figure S2: Specific conductivity vs. concentration curve for C_10_CholBr; Figure S3: Specific conductivity vs. concentration curve for C_12_CholBr; Figure S4: Specific conductivity vs. concentration curve for C_16_CholBr; Figure S5: Number size distribution profiles for aggregates of cholinium-based ILs; Tables S1–S4: raw data on conductivity measurements; Tables S5–S8: raw data on surface tension measurements; Tables S9–S12: raw data on fluorescence measurements; Table S13: intensity size distributions by DLS; Table S14: number size distributions by DLS; Tables S15–S18: raw data on hemolysis percentages. Reference [27] is cited in the Supplementary Materials.
