# Supplementary material for: Cholinium-Based Ionic Liquids as Promising Antimicrobial Agents in Pharmaceutical Applications: Surface Activity, Antibacterial Activity and Ecotoxicological Profile
Source: Pharmaceutics. 2023 Jun 24;15(7):1806. doi: 10.3390/pharmaceutics15071806 (PMC10385515; doi:10.3390/pharmaceutics15071806)
Supplement: Supplementary file 1 [file pharmaceutics-15-01806-s001.zip › pharmaceutics-2441128-supplementary.pdf]

pharmaceutics

MDPI

Supplementary Materials: Cholinium-Based Ionic Liquids as Promising Antimicrobial Agents in Pharmaceutical Applications: Surface Activity, Antibacterial Activity and Ecotoxicological Profile

María Teresa García, Elena Bautista, Ana de la Fuente and Lourdes Pérez

Synthesis Section

Materials

1-bromodecane (98%), 1-bromododecane (97%), 1-bromotetradecane (97%), 1-bromohexadecane (97%), 2-dimethylaminoethanol (≥99.5%) were purchased from Sigma–Aldrich (Darmstadt, Germany). All other chemicals used were of reagent grade. Organic solvents were reagent grade and used without further purification. Deuterated methanol was purchased from Euriso-Top. Water from a Milli-Q Millipore system with electrical resistivity of 18.2 MΩ/cm was used was used to prepare all aqueous solutions.

Synthesis of Cholinium-based Ionic Liquids

General procedure [27]: Alkyl(2-hydroxyethyl)dimethylammonium bromide salts were synthetized by mixing 0,05 mol of 2-dimethylethanolamine with 0.055 mol of the corresponding 1-bromoalkane. 30 mL of 10:1 *v*/*v* acetonitrile/methanol was added to ensure complete miscibility of all starting materials. The mixture was heated at 60 °C and stirred for 30 min. The solvents were evaporated, and the obtained solid product was dissolved in ethyl acetate and cooled. Subsequently, the solid phase was filtered off, and washed two times more with ethyl acetate and a portion of ethyl acetate was added. The obtained bromides were dried under reduced pressure at 40 °C for 48 h and stored over P_4O_{10}.

Analytical data and spectra assignments.

^1H and ^{13}C NMR spectra of the purified products were recorded in CD_3OD (Euriso-Top, Cambridge, UK) on a Varian spectrometer (Palo Alto, California, US) at 400 MHz (^1H) and 101 MHz (^{13}C). HRMS identification of the compounds was performed on an Acquity UPLC System and a LCT Premier™ XE Benchtop orthogonal acceleration TOF (Waters Corporation, Milford, Massachusetts, US) with an electrospray ionization source. Data were processed using MassLynx software (v 4.1). Elemental analysis (CHN) of the final compounds was determined with a Flash*Smart*™ Elemental Analyzer A8 (Thermo Fisher Scientific, Waltham, Massachusetts, US), whereas Br was analyzed by potentiometric titration with AgNO3 with a Metrohm 808 Titrator.

Decyl(2-hydroethyl)dimethylammonium bromide. [C_{10}CholBr] was obtained as a white solid. Yield: 73 93%. ^1H NMR (400 MHz, CD_3OD) δ: 0.89 (t, 3H, $C\underline{H}_3(CH_2)_7CH_2CH_2N^+$); 1.31 (m, 14H, $CH_3(C\underline{H}_2)_7CH_2CH_2N^+$); 1.80 (m, 2H, $CH_3(CH_2)_7C\underline{H}_2CH_2N^+$); 3.17 (s, 6H, $(C\underline{H}_3)_2N^+$); 3.41 (m, 2H, $CH_3(CH_2)_7CH_2C\underline{H}_2N^+$); 3,48 (m, 2H, $N^+C\underline{H}_2CH_2OH$); 3.99 (m, 2H, $N^+CH_2C\underline{H}_2OH$). ^{13}C NMR (101 MHz, CD_3OD) δ: 14.42, 23.65, 23.71, 27.40, 30.22, 30.40, 30.55, 33.03, 52.17, 52.21, 52.24, 56.88, 66.51, 66.82. Anal. calcd. for $C_{14}H_{32}BrNO$: C, 54.2; H, 10.4; N, 4.5; Br, 25.8; Found: C, 52.0; H, 10.4; N, 4.2; Br, 26.1 Found: C, 54.0; H, 10.4; N, 4.4; Br, 25.9. ESI-MS [$C_{10}Chol^+$]: m/z 230.3.

Dodecyl(2-hydroethyl)dimethylammonium bromide. [C_{12}CholBr] was obtained as a white solid. Yield: 57 87%. ^1H NMR (400 MHz, CD_3OD) δ: 0.90 (t, 3H, $C\underline{H}_3(CH_2)_9CH_2CH_2N^+$); 1.31-1,38 (m, 18H, $CH_3(C\underline{H}_2)_9CH_2CH_2N^+$); 1.80 (m, 2H, $CH_3(CH_2)_9C\underline{H}_2CH_2N^+$); 3.17 (s, 6H,

($C\underline{H}_3)_2N^+$); 3.41 (m, 2H, $CH_3(CH_2)_9CH_2C\underline{H}_2N^+$); 3,48 (m, 2H, $N^+C\underline{H}_2CH_2OH$); 3.99 (m, 2H, $N^+CH_2C\underline{H}_2OH$). ^{13}C NMR (101 MHz, MeOD) δ 14.43, 23.65, 23.73, 27.40, 30.22, 30.46, 30.54, 30.63, 30.73, 33.06, 52.17, 52.23, 52.25, 56.88, 66.50, 66.82. Anal. calcd. for $C_{16}H_{36}BrNO$: C, 56.8; H, 10.7; N, 4.1; Br, 23.6; Found: C, 56.9; H, 10.6; N, 4.1; Br, 23.7. ESI-MS [$C_{12}Chol^+$]: m/z 258.3.

Tetradecyl(2-hydroethyl)dimethylammonium bromide. [C_{14}CholBr] was obtained as a white solid. Yield: 78 48%. ^{1}H NMR (400 MHz, CD_3OD) δ: 0.90 (t, 3H, $C\underline{H}_3(CH_2)_{11}CH_2CH_2N^+$); 1.30-1.37 (m, 22H, $CH_3(C\underline{H}_2)_{11}CH_2CH_2N^+$); 1.80 (m, 2H, $CH_3(CH_2)_{11}C\underline{H}_2CH_2N^+$); 3.16 (s, 6H, $(C\underline{H}_3)_2N^+$); 3.41 (m, 2H, $CH_3(CH_2)_{11}CH_2C\underline{H}_2N^+$); 3,48 (m, 2H, $N^+C\underline{H}_2CH_2OH$); 3.99 (m, 2H, $N^+CH_2C\underline{H}_2OH$). ^{13}C NMR (101 MHz, MeOD) δ 14.43, 23.65, 23.73, 27.41, 30.22, 30.47, 30.55, 30.63, 30.72, 30.75, 30.77, 30.79, 33.07, 52.16, 52.20, 56.88, 66.50, 66.83. Anal. calcd. for $C_{18}H_{40}BrNO$: C, 59.0; H, 11.0; N, 3.8; Br, 21.8; Found: C, 58.9; H, 11.0; N, 3.7; Br, 21.9.ESI-MS [$C_{14}Chol^+$]: m/z 286.3.

Hexadecyl(2-hydroethyl)dimethylammonium bromide. [C_{16}CholBr] was obtained as a white solid. Yield: 75 45%. ^{1}H NMR (400 MHz, CD_3OD) δ: 0.90 (t, 3H, $C\underline{H}_3(CH_2)_{13}CH_2CH_2N^+$); 1.19-1.38 (m, 26H, $CH_3(C\underline{H}_2)_{13}CH_2CH_2N^+$); 1.80 (m, 2H, $CH_3(CH_2)_{13}C\underline{H}_2CH_2N^+$); 3.16 (s, 6H, $(C\underline{H}_3)_2N^+$); 3.41 (m, 2H, $CH_3(CH_2)_{13}CH_2C\underline{H}_2N^+$); 3,48 (m, 2H, $N^+C\underline{H}_2CH_2OH$); 3.99 (m, 2H, $N^+CH_2C\underline{H}_2OH$). ^{13}C NMR (101 MHz, MeOD) δ 14.44, 23.65, 23.73, 27.41, 30.23, 30.47, 30.55, 30.64, 30.73, 30.76, 30.78, 30.80, 33.07, 52.20, 56.88, 66.50, 66.83. Anal. calcd. for $C_{20}H_{44}BrNO$: C, 60.9; H, 11.2; N, 3.6; Br, 20.3; Found: C, 60.9; H, 11.3; N, 3.4; Br, 20.3. ESI-MS [$C_{16}Chol^+$]: m/z 314.3.

THERMAL STABILITY

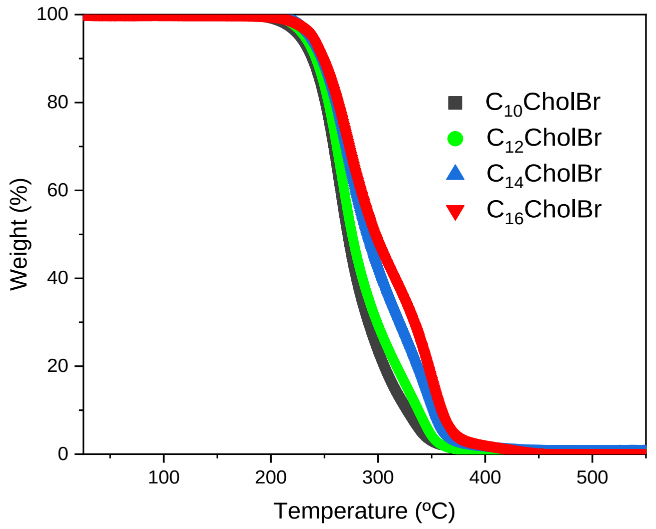

Figure S1. Decomposition curves determined by TGA for C_nCholBr.

Cmc by conductivity measurements

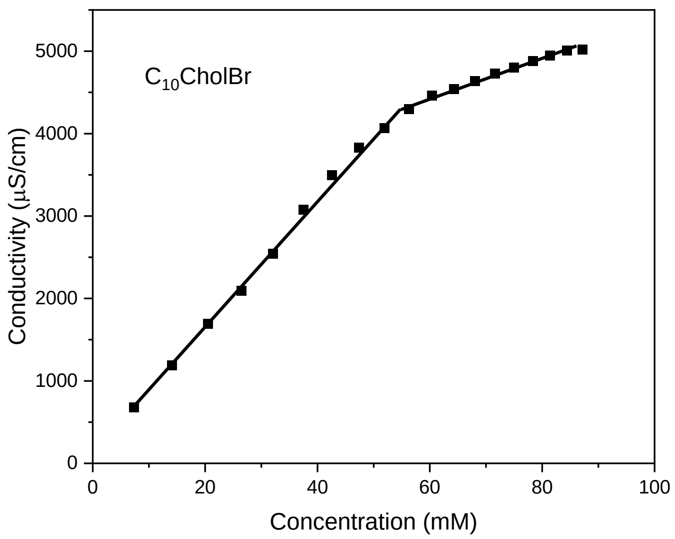

Figure S2. Specific conductivity versus concentration curve for C_{10}CholBr.

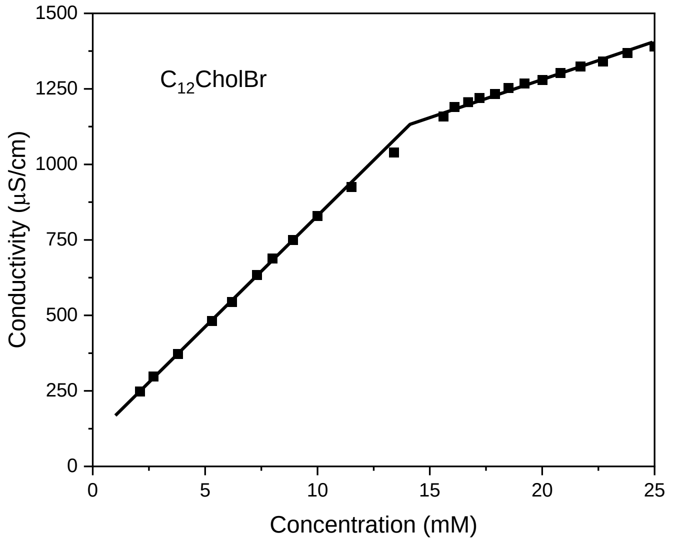

Figure S3. Specific conductivity versus concentration curve for C_{12}CholBr.

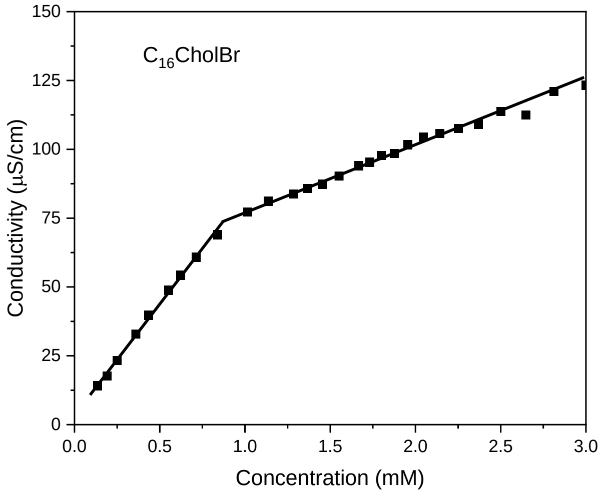

Figure S4. Specific conductivity versus concentration curve for C_{16}CholBr.

DLS measurements

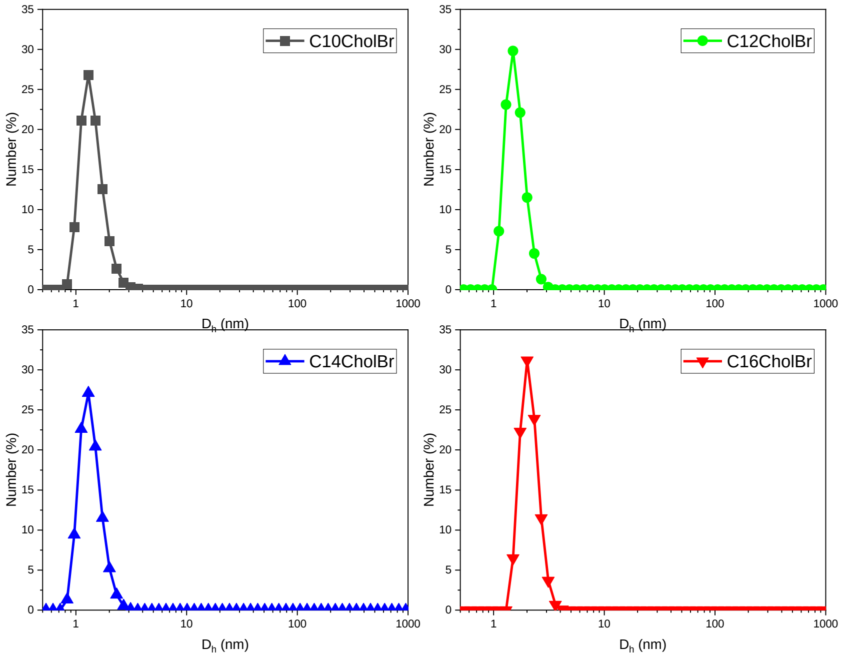

Figure S5. Number size distribution profiles for aggregates of cholinium-based ILs.

Table S1. Cconductivity measurements for C10CholBr.

Concentration (mM)	σ (µS/cm)
7,3	680
14,1	1190
20,5	1693
26,5	2093
32,1	2543
37,5	3080
42,6	3500

47,4	3830
51,9	4070
56,3	4300
60,4	4460
64,3	4540
68	4640
71,6	4730
75	4800
78,3	4880
81,4	4950
84,4	5010
87,2	5020

Table S2. Conductivity measurements for C12CholBr.

**Concentration (mM)**	**σ (μS/cm)**
25,0	1391
23,8	1369
22,7	1341
21,7	1324
20,8	1302
20,0	1280
19,2	1268
18,5	1253
17,9	1234
17,2	1220
16,7	1206
16,1	1190
15,6	1159
13,4	1040
11,5	925
10,0	829
8,9	750
8,0	688
7,3	634
6,2	544
5,3	481
3,8	372
2,7	297
2,1	248

Table S3. Conductivity measurements for C14CholBr.

**Concentration (mM)**	**σ (μS/cm)**
15,0	527
14,1	506
13,2	495
12,5	480
11,8	467
11,3	451
10,7	443
10,2	440
9,8	420
9,4	411
9,0	407
8,7	399
8,3	394
7,8	382
7,3	371
6,8	362
6,12	350
5,56	336
5,1	328
4,37	312
3,83	296

3,09	263
2,6	224
2,24	192
1,97	172
1,27	114
0,93	83,8
0,42	38,3
0,27	24,7
0,20	18,5

Table S4. Conductivity measurements for C16CholBr.

Concentration (mM)	σ (μS/cm)
3,0	123,3
2,81	121,1
2,65	112,5
2,5	113,7
2,37	109,1
2,25	107,5
2,14	105,8
2,05	104,5
1,96	101,7
1,88	98,6
1,8	97,8
1,73	95,4
1,67	94,1
1,55	90,3
1,45	87,4
1,36	85,7
1,29	83,8
1,13	81,2
1,02	77,3
0,84	69
0,71	60,9
0,62	54,3
0,55	48,9
0,44	39,8
0,36	32,9
0,25	23,26
0,19	17,75
0,13	14,24

Table S5. Surface tension measurements for C10CholBr.

Concentration (mM)	Surface Tension (mN/m)
0	72,8
2,39	61,2
4,74	57,9
7,04	51,3
9,30	44,7
13,68	43,0
17,90	37,5
21,97	34,2
29,68	32,0
36,87	31,6
45,19	32,4
52,87	34,7
66,55	40,8
88,72	41,0
105,91	41,0

Table S6. Surface tension measurements for C12CholBr.

Concentration (mM)	Surface Tension (mN/m)
0	72,8

0,73	58,8
1,45	56,4
2,15	52,4
2,84	48,5
4,18	45,8
5,47	44,0
6,72	41,4
9,07	38,3
13,82	38,3
16,16	38,7
20,34	39,0
27,12	38,8
32,38	38,8

Table S7. Surface tension measurements for C14CholBr.

Concentration (mM)	Surface Tension (mN/m)
0,00	72,5
0,24	59,7
0,48	53,0
0,72	50,2
0,94	43,7
1,39	41,7
1,82	40,7
2,23	40,5
3,02	37,2
3,75	37,7
4,59	37,9
6,09	38,1
8,51	37,9
10,36	37,9
11,84	38,0

Table S8. Surface tension measurements for C16CholBr.

Concentration (mM)	Surface Tension (mN/m)
0	72,3
0,015	61.7
0,031	59,5
0,046	57,4
0,076	48,7
0,15	42,7
0,30	38,4
0,43	36,6
0,70	37,4
0,94	36,8
1,22	37,1
1,72	37,0
2,52	37,1
3,13	36,9

Table S9. Fluorescence measurements for C10CholBr.

Concentration (mM)	I_1/I_3
0	1,547
4,3	1,559
8,5	1,546
12,6	1,536
16,6	1,542
20,4	1,551
26,7	1,552
32,7	1,547
43,9	1,568
49,2	1,532
54,3	1,496

64,0	1,300
73,0	1,237
81,4	1,227
89,2	1,232
103	1,227
116	1,213
127	1,240

Table S10. Fluorescence measurements for C12CholBr.

Concentration (mM)	I_1/I_3
0	1,548
2,7	1,561
4,0	1,553
5,3	1,584
7,1	1,545
9,0	1,569
11,3	1,528
13,6	1,397
16,4	1,263
19,0	1,224
21,6	1,209
24,0	1,208
33,0	1,214
37,0	1,210
45,9	1,196

Table S11. Fluorescence measurements for C14CholBr.

Concentration (mM)	I_1/I_3
0	1,596
0,45	1,596
0,89	1,603
1,11	1,599
1,54	1,606
1,97	1,578
2,59	1,602
3,19	1,340
3,78	1,235
4,55	1,241
5,47	1,225
6,35	1,205
7,20	1,220
8,02	1,218
8,80	1,203
15,3	1,235
16,4	1,221
18,3	1,188

Table S12. Fluorescence measurements for C16CholBr.

Concentration (mM)	I_1/I_3
0	1,576
0,05	1,572
0,07	1,573
0,09	1,580
0,13	1,588
0,18	1,575
0,22	1,587
0,32	1,568
0,42	1,571
0,61	1,483
0,78	1,248
1,08	1,231
1,35	1,206

1,58	1,210
1,78	1,197
1,96	1,196

Table S13. DLS: Intensity size distribution of CnCholBr.

Diameter (nm)	C10CholBr	C12CholBr	C14CholBr	C16CholBr
0,4	0	0	0	0
0,4632	0	0	0	0
0,5365	0	0	0	0
0,6213	0	0	0	0
0,7195	0	0	0	0
0,8332	0	0	0	0
0,9649	0,1	0	0,2	0
1,117	2,4	0	2,4	0
1,294	6,5	4,1	6,3	0
1,499	11,3	11,3	10,4	0
1,736	15	17,8	13	1,2
2,01	16,5	19,9	13	4
2,328	15,5	16,6	10,3	6,2
2,696	12,2	9,7	6,1	6,1
3,122	7,8	3,1	2,1	3,6
3,615	3,7	0	0	0,9
4,187	0,9	0	0	0
4,849	0	0	0	0
5,615	0	0	0	0
6,503	0	0	0	0
7,531	0	0	0	0
8,721	0	0	0	0
10,1	0	0	0	0
11,7	0	0	0	0
13,54	0	0	0	0
15,69	0	0	0	0
18,17	0	0	0	0
21,04	0	0	0	0
24,36	0	0	0	0
28,21	0	0	0	0
32,67	0	0	0	0
37,84	0	0	0	0
43,82	0	0	0	0
50,75	0	0	0	0
58,77	0	0	0	0
68,06	0	0	0	0
78,82	0	0,1	0,3	0,6
91,28	0	0,9	1,5	2,7
105,7	0	2,3	3,4	6,3
122,4	0	3,4	5,3	10,4
141,8	0	3,9	6,5	13,7
164,2	0	3,4	6,7	14,8
190,1	0	2,3	5,7	13,3
220,2	0	1	4	9,6
255	0	0,2	2,1	5,1
295,3	0	0	0,6	1,4
342	0	0	0	0
396,1	0	0	0	0
458,7	0	0	0	0
531,2	0	0	0	0
615,1	0	0	0	0
712,4	0,1	0	0	0
825	0,1	0	0	0
955,4	0,2	0	0	0
1106	0,3	0	0	0
1281	0,4	0	0	0
1484	0,4	0	0	0
1718	0,5	0	0	0

1990	0,6	0	0	0
2305	0,6	0	0	0
2669	0,7	0	0	0
3091	0,8	0	0	0
3580	0,8	0	0	0
4145	0,8	0	0	0
4801	0,8	0	0	0
5560	0,8	0	0	0
6439	0	0	0	0
7456	0	0	0	0
8635	0	0	0	0
10000	0	0	0	0

Table S14. DLS: Number size distribution of CnCholBr.

Diameter (nm)	C10CholBr	C12CholBr	C14CholBr	C16CholBr
0,4	0	0	0	0
0,4632	0	0	0	0
0,5365	0	0	0	0
0,6213	0	0	0	0
0,7195	0	0	0	0
0,8332	0,7	0	1,3	0
0,9649	7,8	0	9,4	0
1,117	21,1	7,3	22,6	0
1,294	26,8	23,1	27,1	0
1,499	21,1	29,8	20,4	6,5
1,736	12,6	22,1	11,5	22,3
2,01	6,1	11,5	5,2	31,2
2,328	2,6	4,5	1,9	23,9
2,696	0,9	1,3	0,5	11,5
3,122	0,3	0,3	0,1	3,7
3,615	0,1	0	0	0,7
4,187	0	0	0	0,1
4,849	0	0	0	0
5,615	0	0	0	0
6,503	0	0	0	0
7,531	0	0	0	0
8,721	0	0	0	0
10,1	0	0	0	0
11,7	0	0	0	0
13,54	0	0	0	0
15,69	0	0	0	0
18,17	0	0	0	0
21,04	0	0	0	0
24,36	0	0	0	0
28,21	0	0	0	0
32,67	0	0	0	0
37,84	0	0	0	0
43,82	0	0	0	0
50,75	0	0	0	0
58,77	0	0	0	0
68,06	0	0	0	0
78,82	0	0	0	0
91,28	0	0	0	0
105,7	0	0	0	0
122,4	0	0	0	0
141,8	0	0	0	0
164,2	0	0	0	0
190,1	0	0	0	0
220,2	0	0	0	0
255	0	0	0	0
295,3	0	0	0	0
342	0	0	0	0
396,1	0	0	0	0
458,7	0	0	0	0

531,2	0	0	0	0
615,1	0	0	0	0
712,4	0	0	0	0
825	0	0	0	0
955,4	0	0	0	0
1106	0	0	0	0
1281	0	0	0	0
1484	0	0	0	0
1718	0	0	0	0
1990	0	0	0	0
2305	0	0	0	0
2669	0	0	0	0
3091	0	0	0	0
3580	0	0	0	0
4145	0	0	0	0
4801	0	0	0	0
5560	0	0	0	0
6439	0	0	0	0
7456	0	0	0	0
8635	0	0	0	0
10000	0	0	0	0

Table S15. Percentage of hemolysis vs concentration for C12CholBr.

Concentration (μg/mL)	Hemolysis (%)
150	−0,69
300	−0,32
450	−0,77
600	−0,37
750	−0,21
900	0,89
1050	11,0
1200	37,4
1350	78,5
1500	97,6
2500	99,0

Table S16. Percentage of hemolysis vs concentration for C14CholBr.

Concentration (μg/mL)	Hemolysis (%)
12,5	0,56
25	0,82
37,5	0,49
50	0,65
62,5	1,0
75	5,2
87,5	22,2
100	74,9
112,5	96,6
125	94,2
250	99,0

Table S17. Percentage of hemolysis vs concentration for C16CholBr.

Concentration (μg/mL)	Hemolysis (%)
5	1,5
10	2,7
20	15,0
30	65,0
40	92,2
50	99,3
60	99,3
70	100,4
80	99,8
90	98,0

100	101

References

27. Garcia, M.T.; Ribosa, I.; Perez, L.; Manresa, A.; Comelles, F. Aggregation Behavior and Antimicrobial Activity of Ester-Functionalized Imidazolium- and Pyridinium-Based Ionic Liquids in Aqueous Solution. *Langmuir* **2013**, *29*, 2536–2545. https://doi.org/10.1021/la304752e.
